# Late-onset-case of Familial Mediterranean fever (FMF)—exotic but rising differential diagnosis of “unclear abdominal illness” in Germany due to increasing migration over the past decade

**DOI:** 10.1007/s10354-025-01114-7

**Published:** 2025-11-07

**Authors:** M. R. Kanaan, F. Meyer

**Affiliations:** 1 Department of General, Abdominal, Vascular and Transplant Surgery, Otto-von-Guericke University Medical School, Leipziger Str. 44, 39120 Magdeburg, Germany; 2 Department of Urology and Urologic Oncology, Hannover Medical School, Hannover, Germany

**Keywords:** Fever and abdominal pain, Acute abdomen, Differential diagnosis, Medical history, Abdominal symptoms, Fieber und Bauchschmerz, Akutes Abdomen, Differentialdiagnose, Anamnese, Abdominalsymptome

## Abstract

**Background:**

Recurrent fever and acute abdominal pain are common reasons for emergency visits, particularly in adolescents. These symptoms often lead to misdiagnosis and unnecessary surgeries like appendectomy. Familial Mediterranean fever (FMF), a rare autoinflammatory disorder, should be considered in patients with periodic febrile abdominal episodes, especially in those with a migration background.

**Case presentation:**

A 19-year-old male of Syrian origin presented with acute right lower quadrant abdominal pain, fever, arthralgia, and pleuritic chest pain. Similar episodes had occurred in the past and resolved spontaneously. Physical examination revealed signs of peritonism; laboratory results showed elevated C‑reactive protein (CRP; 35 mg/L) with normal white blood cell counts. Abdominal ultrasound excluded appendicitis. Conservative treatment (fluids, analgesics, dietary rest) led to full symptom resolution within 2 days. After recovery, a detailed family history revealed consanguinity and a relative with similar symptoms. The diagnosis of FMF was made based on the Tel Hashomer criteria. Colchicine therapy (1.0 mg/day) was initiated.

**Conclusion:**

This report illustrates a case of late-onset FMF in a migrant adolescent, with typical symptoms but without definitive serositis or confirmed genetic diagnosis at the time of treatment. The case underscores the importance of thorough history-taking and highlights the need to avoid unnecessary CT scans in similar settings. Awareness of FMF is crucial in nonendemic regions experiencing increased migration from high-incidence countries.

## Abstract (de)

**Hintergrund:**

Wiederkehrendes Fieber und akute Bauchschmerzen gehören zu den häufigen Vorstellungsgründen in der Notaufnahme, insbesondere bei Jugendlichen. Diese Symptome führen nicht selten zu Fehldiagnosen und unnötigen chirurgischen Eingriffen wie einer Appendektomie. Das familiäre Mittelmeerfieber (FMF), eine seltene autoinflammatorische Erkrankung, sollte bei Patienten mit periodischen febrilen Bauchbeschwerden – v. a. mit Migrationshintergrund – differenzialdiagnostisch berücksichtigt werden.

**Fallbericht:**

Ein 19-jähriger Mann syrischer Herkunft stellte sich mit akuten Schmerzen im rechten Unterbauch, Fieber, Arthralgien und atemabhängigen Thoraxschmerzen vor. Ähnliche Episoden waren in der Vergangenheit spontan abgeklungen. Klinisch zeigten sich Zeichen eines Peritonismus, laborchemisch ein erhöhter Level für C‑reaktives Protein (CRP; 35 mg/l) bei normalen Leukozyten. Sonographisch ergaben sich keine Hinweise auf eine Appendizitis. Unter konservativer Therapie (Infusionen, Analgetika, Nahrungskarenz) kam es innerhalb von 2 Tagen zur vollständigen Besserung. Eine ausführliche Familienanamnese offenbarte eine Konsanguinität und einen ähnlichen Fall in der Familie. Die Diagnose FMF wurde anhand der Tel-Hashomer-Kriterien gestellt. Eine Therapie mit Colchicin (1,0 mg/Tag) wurde begonnen.

**Schlussfolgerung:**

Der Fall zeigt eine Spätmanifestation des FMF bei einem jugendlichen Migranten mit typischer Klinik, jedoch ohne nachgewiesene Serositis oder genetische Bestätigung zum Zeitpunkt der Behandlung. Er verdeutlicht die Bedeutung einer sorgfältigen Anamnese und spricht gegen den routinemäßigen Einsatz der Computertomographie(CT)-Diagnostik bei ähnlicher Konstellation. Das Bewusstsein für FMF sollte auch in Ländern mit niedriger Prävalenz erhöht werden.

## Introduction

Familial Mediterranean fever (FMF) is a hereditary autoinflammatory disorder caused by mutations in the *MEFV* gene and leading to dysregulation of the inflammasome and subsequent inflammatory attacks. Typical manifestations include recurrent episodes of fever, abdominal pain, and pleurisy [1, 2]. However, a lack of knowledge of and attention to FMF leads often to delayed diagnosis and management, especially in countries with low disease prevalence and incidence rates [3].

This paper reports the case of an adolescent patient with FMF who presented with a late disease manifestation, emphasizing the need for awareness of FMF, especially as the migration of people from high-incidence areas to Germany has been increasing for the past few years.

## Case summary

### Medical history

A 19-year-old male patient presented to the emergency department because of an episode of fever accompanied by acute abdominal pain in the right lower abdomen for 2 days with worsening tendency. Other symptoms were pain due to arthralgia and thoracic pain during breathing. The patient had a medical history of similar episodes in the past, with complete remission after 2 to 3 days without any specific treatment.

At admission, the patient reported no previous medication or surgical intervention. Regarding social and family history, the patient was a student and had grown up in Syria. He had been in Germany for 3 years.

### Physical examination

The patient was in severely reduced general condition, with normal nutritional status. He had stable vital signs and there was no disturbance of consciousness. Examination of the abdomen revealed marked tenderness in the lower right quadrant. The abdominal wall was rigid, and there were signs of peritonism.

### Diagnostic measures

Laboratory values upon admission showed inflammatory signs, with a serum C‑reactive protein (CRP) level of 35.0 mg/L (normal range < 5 mg/L). The white blood cell count was within the normal range at 8.6 GpT/L (normal range < 10 GpT/L). Another routine profile of laboratory parameters was within normal limits. Serological tests for common infections were negative.

Abdominal ultrasound revealed no signs of appendicitis or other serious diseases or pathological findings.

A pleural ultrasound was not performed; however, the clinical presentation of pleuritic chest pain is consistent with serositis. No definitive serosal effusion was confirmed.

### Suspicious diagnosis

- Recurrent (periodic) abdominal pain with fever not-other specified.

### Differential diagnoses

The spectrum of differential diagnoses comprised acute abdominal diseases like appendicitis, diverticulitis, and other hereditary autoinflammatory disorders with fever coexisting with abdominal pain in adolescents and children, such as tumor necrosis factor receptor-associated periodic syndrome (TRAPS)/hyperimmunoglobulin‑D syndrome (HIDS; a very rare autoinflammatory disease belonging to the periodic fever syndromes), or FMF.

### Differential therapeutic approach

Considering the similar episodes in the past, the indication for a conservative therapeutic approach with infusion therapy, administration of analgesics, “n. p.o.” (initial abstinence from food), and a restorative diet was given.

### Diagnosis and management

Retaking the patient’s family medical history after convalescence revealed similar cases in the family. It was discovered that the patient’s parents were related, and there had been a similar case in the family with the same symptoms 2 years ago.

Clinical findings resulted in a diagnosis of FMF based on clinical presentation and fulfilment of the Tel Hashomer criteria (Table 1). Further genetic testing was not performed.

**Table 1:** Simplified Tel Hashomer criteria for the diagnosis of Familial Mediterranean fever (FMF; modified from [7])

Major criteria	Minor criteria
1) Recurrent febrile attacks accompanied by peritonitis, synovitis, or pleuritis 2) Amyloidosis of the AA type without predisposing disease 3) Favorable response to continuous colchicine treatment	1) Recurrent febrile attacks 2) Erysipelas-like erythema 3) FMF in a first-degree relative

### Definitive diagnosis

- Familial Mediterranean fever (FMF).

### Treatment

Treatment with 1.0 mg colchicine per day was initiated as the gold standard in disease management to prevent renal and cardiac amyloidosis and which was done to achieve prompt and adequate medical attention for (also further episodes) fever and serositis.

### Further advice/procedure

- Treatment with colchicine should be maintained to reduce the frequency and intensity of febrile episodes and pain during attacks.
- A follow-up control of laboratory parameters in addition to assessing the serum level of amyloid AA during acute episodes should be favored. Furthermore, regular follow-up care by a rheumatologist or nephrologist should be established to monitor therapy. Adequate information about the side effects of colchicine should be provided to maintain patient care.
- Genetic testing for FMF can be conducted if the test is available.

## Discussion

**Fig. 1:**
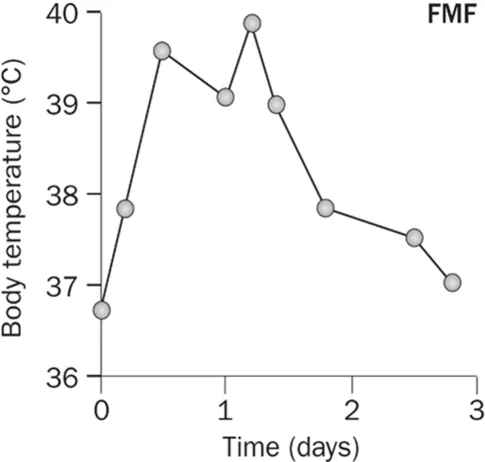
Patterns of fever seen in patients with familial Mediterranean fever (*FMF*) [14]

Familial Mediterranean fever is a typically autosomal recessive condition that arises due to a mutation in the *MEFV* gene. This gene encodes a protein called pyrin, which plays a crucial role in regulating the inflammatory process within the body. Familial Mediterranean fever has been associated with over 80 different mutations in the *MEFV* gene, leading to an abnormal inflammatory response and resulting in recurrent episodes of serositis. Patients with FMF frequently experience fever with a typical fever pattern (Fig. 1), peritonitis, synovitis, pleurisy, arthritis, and occasionally pericarditis [1].

While FMF can affect any population, it is more prevalent among individuals of Arabic, North African Jewish, Armenian, and Turkish descent. In specific populations, the estimated prevalence can be as high as 1 in 7 individuals [1, 2].

In the current case, the patient presented with the classical manifestation of “periodic peritonitis,” which occurs in up to 90% of affected individuals [1]. Unfortunately, these clinical features often lead to unnecessary diagnostic laparoscopy or laparotomy, as common surgical conditions like appendicitis, bowel obstruction, and cholecystitis might be mistaken as causes of the symptoms. Kasifoglu et al. reported a significantly higher rate of appendectomy in FMF patients before their diagnosis compared to a healthy control population (26.7% vs. 4.9%; *p* = 0.0001) [3].

The average age of onset in FMF is typically in childhood, with most patients experiencing first symptoms before the age of 10 years. Therefore, the 19-year-old patient in this case represents a late-onset presentation, which is less common but well documented in the literature [5].

This case underscores the significance of identifying this uncommon condition in patients of Mediterranean descent who present with acute abdomen, especially children and adolescents.

The described patient presented at the age of 19 years but had been experiencing symptoms for 2 years. This form of clinical course emphasizes the need for early recognition and diagnosis in such cases. The absence of precise prevalence data and awareness of this condition in countries with FMF as a rare disease might lead to delays in diagnosis and poorer patient outcomes. In Germany, there are limited data available regarding the distribution, ethnic backgrounds, and accurate prevalence of FMF patients [4, 6], although approximately 10% of the general population in Germany has Mediterranean roots or has migrated from the area of the Silk Road, potentially putting them at risk of developing FMF [4]. Indeed, this proportion is on the rise due to increasing migration in recent years. The growing number of people with Mediterranean roots may lead to a higher incidence and prevalence in the next years.

The clinical diagnosis of FMF can be established using the Tel Hashomer criteria (Table 1). According to these criteria, either two major criteria (fever and serositis, amyloidosis AA, effectiveness of colchicine) or one major and two minor criteria (recurrent attacks of fever, erysipelas-like erythema, relatives affected by FMF) must be present to make the diagnosis.

However, it is important to note that genetic testing for FMF has a positive predictive value of only 70%–80%, meaning that it may not always provide definitive confirmation of the disease. Therefore, a combination of clinical criteria and genetic testing is typically used for a more accurate diagnosis [7].

A notable and extensively documented long-term complication of FMF is AA amyloidosis. Amyloidosis is a multisystem disorder resulting from the aggregation of amyloid protein. In individuals with FMF, these amyloid deposits frequently accumulate in the kidneys, leading to the development of nephrotic syndrome and ultimately progressing to end-stage renal failure [1, 2]. According to a retrospective cohort study involving more than 1000 participants with FMF, renal amyloidosis accounted for 35% and 60% of deaths in men and women with FMF, respectively [9].

**Fig. 2:**
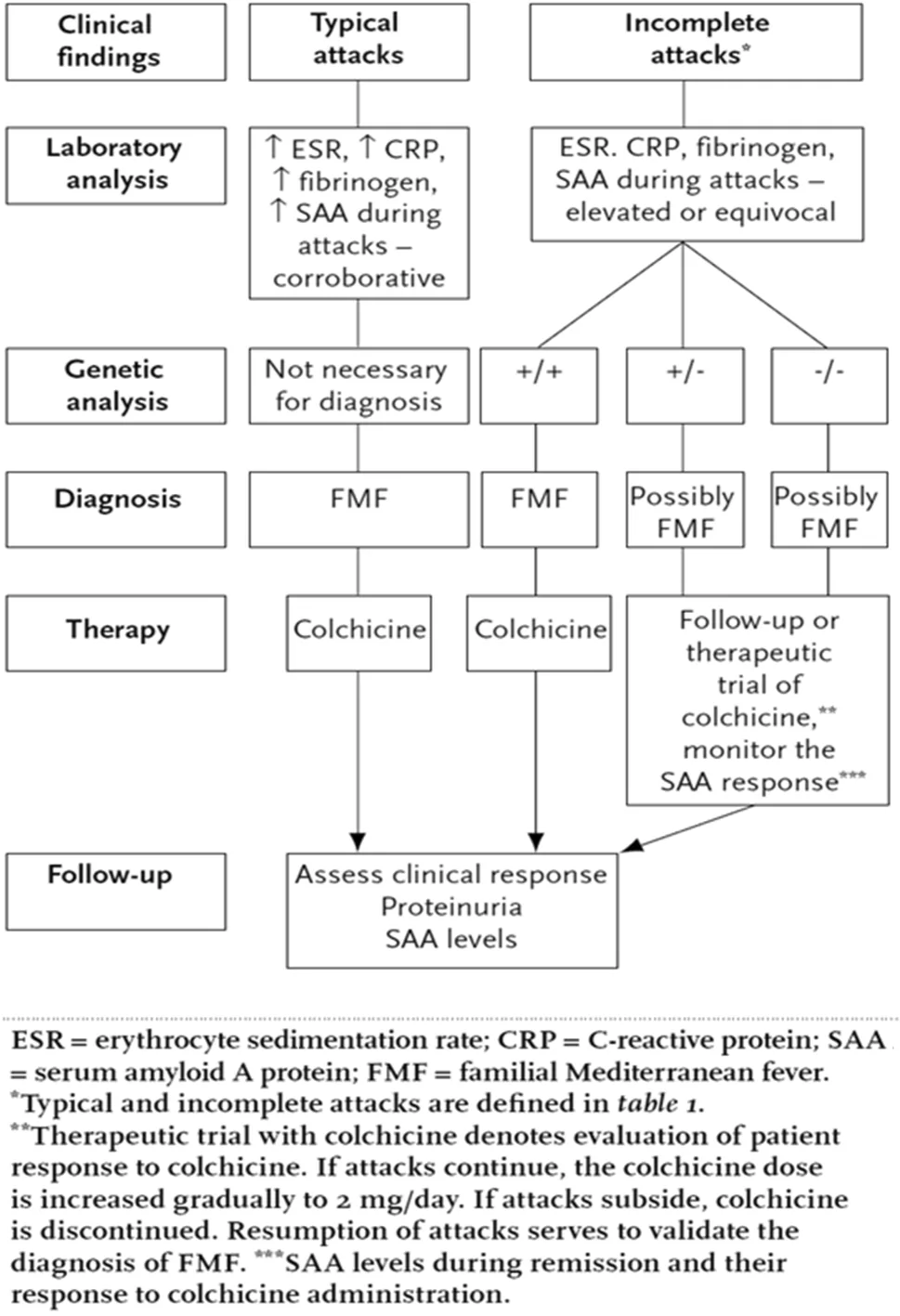
Diagnostic algorithm for clinical application in a patient with familial Mediterranean fever (*FMF*)

Relying solely on clinical assessment for diagnosis poses the risk of overlooking other pathologies, such as appendicitis, and imaging can aid in excluding alternative diagnoses. While imaging is not part of the diagnostic criteria for FMF (Fig. 2), it is recommended to use it to rule out other conditions that might coexist or present with similar symptoms. This approach helps to ensure a more accurate diagnosis and appropriate management of the patient’s condition.

Colchicine has been the gold standard primary treatment for FMF since the 1970s, and there is evidence suggesting that it may reduce the risk of progression to amyloidosis [10]. Colchicine is highly effective in preventing FMF attacks and providing long-term relief for patients, leading to improved symptom management and a better quality of life [10]. Common side effects of colchicine treatment encompass diarrhea, vomiting, and abdominal pain. Fortunately, these adverse effects can often be managed effectively by either lowering the dosage or prescribing smaller doses administered twice daily. However, it is crucial to remain vigilant, as gastrointestinal symptoms may also signal the initial stages of colchicine toxicity. In such cases, prompt medical attention is necessary to ensure the safe and appropriate use of the medication. Regular monitoring and open communication with healthcare providers are essential to minimize the risks associated with colchicine therapy and to optimize patient outcomes [2, 11].

The guideline issued by the German Society for Paediatric Rheumatology dates back to 2013. More contemporary guidance—most prominently the 2024 European Alliance of Associations for Rheumatology (EULAR)/Paediatric Rheumatology European Society (PReS)-endorsed recommendations—provides an evidence-based foundation for clinical decision-making [10, 12]. These international recommendations confirm colchicine as the first-line disease-modifying agent and advocate early introduction of interleukin‑1 inhibitors (anakinra or canakinumab) in patients who are either resistant or intolerant to colchicine. While *MEFV* (Mediterranean fever) genotyping is encouraged, it is not mandatory when validated clinical criteria are satisfied.

However, in cases in which patients show an ineffective response to colchicine or experience intolerable side effects, biologics targeting interleukin(IL)-1 have been used as an alternative treatment. Among these biologics, canakinumab is one such inhibitor [10, 12].

Additionally, besides canakinumab, another IL‑1 inhibitor, anakinra, has also demonstrated positive effects in FMF management. Studies have shown that anakinra can reduce the frequency of symptoms, normalize inflammatory markers, and limit the progression of renal amyloidosis in affected patients. These biological therapies represent important treatment options for individuals who do not respond well to colchicine or experience adverse effects [12, 13].

The current patient presented with typical symptoms of FMF, with fever associated with abdominal and thoracic pain. In this specific case, the diagnosis was confirmed by clinical criteria according to the Tel Hashomer criteria.

Importantly, no abdominal CT scan was performed in this case. The use of abdominal CT should be avoided whenever possible in young patients with suspected FMF due to the risk of unnecessary radiation exposure and the often non-specific imaging findings in FMF. Clinical evaluation supported by ultrasound and laboratory markers is generally sufficient for diagnostic orientation.

As a limitation, so far there are predominantly single cases reported in the literature. In addition, the majority of the available studies are retrospective case series with no control group. However, the approach to the clinical care of the presented case may result in valuable clinical experiences in diagnosis-related case management.

## Conclusion

Familial Mediterranean fever (FMF) is recognized as an exotic but increasingly diagnosed condition in Germany, primarily attributed to the rise in migration observed over the past decade. The growing number of individuals with Mediterranean roots or migrating from affected regions may contribute to the higher incidence of FMF cases in the country.

Raising awareness of the disease symptoms, particularly among children with a migration background, is crucial. Increasing knowledge about FMF and its presentation is essential to ensure early detection and appropriate management. As FMF is the most common autoimmune inflammatory disease, improving its management is of utmost importance to provide better healthcare outcomes for affected individuals.
